# Bis[μ-2-(2-naphth­oxy)acetato]bis­{aqua[2-(2-naphth­oxy)acetato]zinc(II)}

**DOI:** 10.1107/S1600536809013750

**Published:** 2009-04-22

**Authors:** Fang-Jun Chen, Hai Xu, Hui Xu, Ke-Long Huang

**Affiliations:** a Department of Chemistry, Central South University, Changsha 410083, People’s Republic of China

## Abstract

The title binuclear Zn^II^ compound, [Zn_2_(C_12_H_9_O_3_)_4_(H_2_O)_2_], is centrosymmetric. Each Zn atom is coordinated by two bridging 2-naphthoxyacetate anions, one terminal 2-naphth­oxy­acetate anion and one water mol­ecule in a distorted ZnO_4_ tetra­hedral geometry. The naphthalene system of the bridging ligand is nearly perpendicular to the naphthalene of the terminal ligand, with a dihedral angle of 78.26 (6)°. Within the binuclear mol­ecule the Zn⋯Zn separation is 3.815 (5) Å. In the crystal structure, inter­molecular O—H⋯O hydrogen bonding between the water mol­ecule and carboxyl­ate groups helps to stabilize the crystal structure.

## Related literature

For general background, see: Harrison *et al.* (2002 ▶); Ma *et al.* (2004 ▶). For a related structure, see: Li *et al.* (2008 ▶).
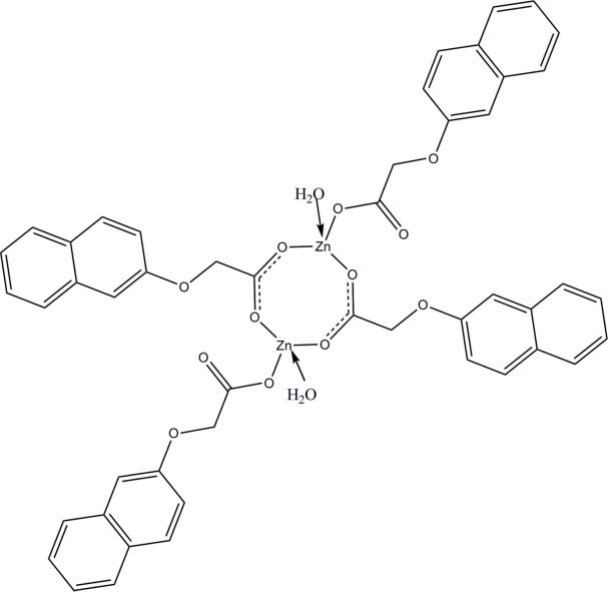


## Experimental

### 

#### Crystal data

[Zn_2_(C_12_H_9_O_3_)_4_(H_2_O)_2_]
*M* *_r_* = 971.54
Triclinic, 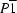

*a* = 5.3241 (5) Å
*b* = 9.2189 (10) Å
*c* = 20.722 (2) Å
α = 86.055 (6)°
β = 89.351 (6)°
γ = 89.277 (6)°
*V* = 1014.54 (18) Å^3^
*Z* = 1
Mo *K*α radiation
μ = 1.26 mm^−1^
*T* = 296 K
0.35 × 0.19 × 0.10 mm

#### Data collection

Bruker APEXII area-detector diffractometer
Absorption correction: multi-scan (*SADABS*; Sheldrick, 1996 ▶) *T*_min_ = 0.75, *T*_max_ = 0.88
17599 measured reflections
4580 independent reflections
4038 reflections with *I* > 2σ(*I*)
*R*_int_ = 0.024

#### Refinement

*R*[*F* ^2^ > 2σ(*F* ^2^)] = 0.026
*wR*(*F* ^2^) = 0.068
*S* = 1.04
4580 reflections
295 parameters
5 restraints
H atoms treated by a mixture of independent and constrained refinement
Δρ_max_ = 0.37 e Å^−3^
Δρ_min_ = −0.24 e Å^−3^

### 

Data collection: *APEX2* (Bruker, 2006 ▶); cell refinement: *SAINT* (Bruker, 2006 ▶); data reduction: *SAINT*; program(s) used to solve structure: *SHELXTL* (Sheldrick, 2008 ▶); program(s) used to refine structure: *SHELXTL*; molecular graphics: *SHELXTL*; software used to prepare material for publication: *SHELXTL*.

## Supplementary Material

Crystal structure: contains datablocks I, global. DOI: 10.1107/S1600536809013750/xu2494sup1.cif

Structure factors: contains datablocks I. DOI: 10.1107/S1600536809013750/xu2494Isup2.hkl

Additional supplementary materials:  crystallographic information; 3D view; checkCIF report

## Figures and Tables

**Table 1:** Selected bond lengths (Å)

Zn1—O2^i^	1.9492 (12)
Zn1—O3	2.0143 (12)
Zn1—O6	1.9567 (11)
Zn1—O1*W*	1.9496 (12)

Symmetry code: (i) 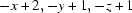.

**Table 2:** Hydrogen-bond geometry (Å, °)

*D*—H⋯*A*	*D*—H	H⋯*A*	*D*⋯*A*	*D*—H⋯*A*
O1*W*—H1*WA*⋯O5^ii^	0.821 (15)	1.810 (15)	2.6284 (18)	174 (2)
O1*W*—H1*WB*⋯O1^iii^	0.791 (15)	2.53 (2)	3.1087 (17)	131 (2)
O1*W*—H1*WB*⋯O3^iii^	0.791 (15)	2.122 (17)	2.8724 (16)	158 (2)

Symmetry codes: (ii) 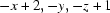; (iii) 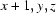.

**Fig. 1.**
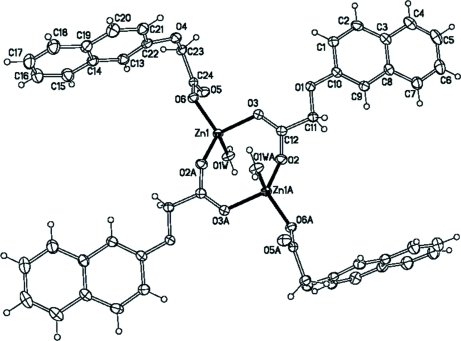
The molecular structure of the title compound, showing 30% probability displacement ellipsoids [symmetry code: (A) -x + 2, -y + 1, -z + 1].

## supplementary crystallographic information

### Comment

The synthesis of metal–organic hybrid materials has been deeply researched as their interesting structural diversity and potential functions (Harrison *et al.*, 2002). In particular, carboxylate ligands, especially aromatic carboxylate ligands, have been shown to be good building blocks in the synthesis of metal–organic materials with desired topologies, owing to their rich coordination modes. The coordination chemistry of flexible aromatic carboxylic acids such as 2-naphthoxyacetic acid (Ma *et al.*, 2004) has captured the attention of chemist for many years. Herein we report the crystal structure of the title compound incorporating 2-naphthoxyacetate ligands.

The binuclear molecule of the title complex is centrosymmetric. The coordination environment of the Zn atom displays a distorted ZnO_4_ tetrahedron (Fig. 1). The molecule contains two Zn atoms connected by two bridge 2-naphthoxyacetate anions and two terminal 2-naphthoxyacetate anions and two coordinate water molecules. Within the binuclear molecule the dihedral angle between bridge naphthalene ring systems is 1.77 (3)°, and that between terminal naphthalene systems is 2.59 (2)°. The C1-containg naphthalene is nearly perpendicular to the C13-containig naphthalene with a dihedral angle of 78.26 (6)°. The bond angles at the Zn center range from 95.10 (5)° to 138.94 (5)°. The coordinate bond distances (Table 1) range from 1.949 (3) to 2.014 (4) Å, which are comparable to those found in a Zn^II^ complex (Li *et al.*, 2008). Within the binuclear molecule the Zn···Zn distance is 3.815 (5) Å.

In the crystal structure there are intermolecular O—H···O hydrogen bonds between water O and 2-naphthoxyacetate O atoms (Table 2), which helps to stabilize the crystal structure.

### Experimental

A mixture of Zn(CH_3_COO)_2_.2H_2_O (0.2195 g, 1 mmol), NaOH (0.021 g, 0.5 mmol), 2-naphthoxyacetic acid (0.202 g, 1 mmol), 2,2'-bipyridine (0.078 g, 0.5 mmol) was dissolved in 17 ml of 15:2 water/ethanol. The solution was placed in a 25 ml Teflon-lined stainless steel bomb. The bomb was heated to 433 K for 3 d. Then it was cooled to room temperature over 3 d. The colorless crystals of the title compound suitable for X-ray diffraction structure analysis were isolated from the solution.

### Refinement

The carbon-bound H atoms were positioned geometrically and included in the refinement using a riding model with C—H = 0.93 Å for aromatic and C—H = 0.97 Å for the others, and *U*_iso_(H) = 1.2*U*_eq_(C). The water H atoms were located from a different map and their positions were refined with restraints of O—H = 0.80 (2) Å and H···H = 1.30 (2) Å, their displacement parameters were set to 1.5*U*_eq_(O).

### Crystal data

**Table:**

[Zn_2_(C_12_H_9_O_3_)_4_(H_2_O)_2_]	*Z* = 1
*M**_r_* = 971.54	*F*(000) = 500
Triclinic, *P*1	*D*_x_ = 1.590 Mg m^−^^3^
Hall symbol: -P 1	Mo *K*α radiation, λ = 0.71073 Å
*a* = 5.3241 (5) Å	Cell parameters from 6935 reflections
*b* = 9.2189 (10) Å	θ = 2.0–27.7°
*c* = 20.722 (2) Å	µ = 1.26 mm^−^^1^
α = 86.055 (6)°	*T* = 296 K
β = 89.351 (6)°	Block, colourless
γ = 89.277 (6)°	0.35 × 0.19 × 0.10 mm
*V* = 1014.54 (18) Å^3^	

### Data collection

**Table:**

Bruker APEXII area-detector diffractometer	4580 independent reflections
Radiation source: fine-focus sealed tube	4038 reflections with *I* > 2σ(*I*)
graphite	*R*_int_ = 0.024
ω scans	θ_max_ = 27.7°, θ_min_ = 2.0°
Absorption correction: multi-scan (*SADABS*; Sheldrick, 1996)	*h* = −6→6
*T*_min_ = 0.75, *T*_max_ = 0.88	*k* = −11→11
17599 measured reflections	*l* = −26→26

### Refinement

**Table:**

Refinement on *F*^2^	Primary atom site location: structure-invariant direct methods
Least-squares matrix: full	Secondary atom site location: difference Fourier map
*R*[*F*^2^ > 2σ(*F*^2^)] = 0.026	Hydrogen site location: inferred from neighbouring sites
*wR*(*F*^2^) = 0.068	H atoms treated by a mixture of independent and constrained refinement
*S* = 1.04	*w* = 1/[σ^2^(*F*_o_^2^) + (0.0351*P*)^2^ + 0.239*P*] where *P* = (*F*_o_^2^ + 2*F*_c_^2^)/3
4580 reflections	(Δ/σ)_max_ = 0.001
295 parameters	Δρ_max_ = 0.37 e Å^−^^3^
5 restraints	Δρ_min_ = −0.24 e Å^−^^3^

### Special details

**Table:**

Geometry. All e.s.d.'s (except the e.s.d. in the dihedral angle between two l.s. planes) are estimated using the full covariance matrix. The cell e.s.d.'s are taken into account individually in the estimation of e.s.d.'s in distances, angles and torsion angles; correlations between e.s.d.'s in cell parameters are only used when they are defined by crystal symmetry. An approximate (isotropic) treatment of cell e.s.d.'s is used for estimating e.s.d.'s involving l.s. planes.
Refinement. Refinement of *F*^2^ against ALL reflections. The weighted *R*-factor *wR* and goodness of fit *S* are based on *F*^2^, conventional *R*-factors *R* are based on *F*, with *F* set to zero for negative *F*^2^. The threshold expression of *F*^2^ > σ(*F*^2^) is used only for calculating *R*-factors(gt) *etc*. and is not relevant to the choice of reflections for refinement. *R*-factors based on *F*^2^ are statistically about twice as large as those based on *F*, and *R*- factors based on ALL data will be even larger.

### Fractional atomic coordinates and isotropic or equivalent isotropic displacement parameters (Å^2^)

**Table:**

	*x*	*y*	*z*	*U*_iso_*/*U*_eq_	
Zn1	0.92519 (3)	0.305896 (19)	0.534483 (9)	0.03119 (7)	
C1	0.1095 (3)	0.23704 (19)	0.30443 (9)	0.0404 (4)	
H1A	0.0692	0.1910	0.3445	0.048*	
C2	−0.0242 (3)	0.2083 (2)	0.25138 (10)	0.0450 (4)	
H2A	−0.1567	0.1435	0.2558	0.054*	
C3	0.0341 (3)	0.27465 (19)	0.18985 (9)	0.0402 (4)	
C4	−0.0984 (4)	0.2444 (2)	0.13329 (11)	0.0553 (5)	
H4A	−0.2325	0.1806	0.1364	0.066*	
C5	−0.0309 (4)	0.3078 (3)	0.07485 (11)	0.0633 (6)	
H5A	−0.1187	0.2870	0.0381	0.076*	
C6	0.1696 (4)	0.4039 (3)	0.06943 (10)	0.0588 (6)	
H6A	0.2154	0.4461	0.0290	0.071*	
C7	0.2989 (4)	0.4367 (2)	0.12279 (10)	0.0498 (5)	
H7A	0.4303	0.5023	0.1185	0.060*	
C8	0.2361 (3)	0.37261 (19)	0.18439 (9)	0.0370 (4)	
C9	0.3695 (3)	0.40425 (19)	0.24049 (9)	0.0379 (4)	
H9A	0.4985	0.4715	0.2373	0.045*	
C10	0.3091 (3)	0.33636 (17)	0.29899 (8)	0.0333 (3)	
C11	0.6150 (3)	0.46390 (17)	0.35489 (8)	0.0326 (3)	
H11A	0.5344	0.5586	0.3482	0.039*	
H11B	0.7322	0.4521	0.3194	0.039*	
C12	0.7539 (3)	0.45536 (16)	0.41740 (8)	0.0305 (3)	
C13	0.4572 (3)	0.09687 (19)	0.74494 (9)	0.0382 (4)	
H13A	0.5845	0.0400	0.7282	0.046*	
C14	0.4585 (3)	0.12649 (19)	0.81109 (9)	0.0389 (4)	
C15	0.6409 (4)	0.0649 (2)	0.85421 (10)	0.0523 (5)	
H15A	0.7679	0.0061	0.8387	0.063*	
C16	0.6335 (4)	0.0904 (3)	0.91819 (11)	0.0605 (6)	
H16A	0.7537	0.0480	0.9459	0.073*	
C17	0.4455 (5)	0.1803 (3)	0.94243 (11)	0.0612 (6)	
H17A	0.4420	0.1973	0.9862	0.073*	
C18	0.2679 (4)	0.2428 (2)	0.90229 (10)	0.0547 (5)	
H18A	0.1443	0.3024	0.9189	0.066*	
C19	0.2695 (3)	0.2181 (2)	0.83567 (9)	0.0416 (4)	
C20	0.0853 (4)	0.2775 (2)	0.79250 (9)	0.0457 (4)	
H20A	−0.0365	0.3404	0.8076	0.055*	
C21	0.0821 (3)	0.24504 (19)	0.72961 (9)	0.0402 (4)	
H21A	−0.0426	0.2840	0.7023	0.048*	
C22	0.2692 (3)	0.15170 (18)	0.70574 (8)	0.0333 (3)	
C23	0.4052 (3)	0.02700 (17)	0.61316 (8)	0.0339 (3)	
H23A	0.3199	−0.0211	0.5796	0.041*	
H23B	0.4617	−0.0473	0.6453	0.041*	
C24	0.6312 (3)	0.10515 (17)	0.58379 (7)	0.0292 (3)	
O1W	1.2112 (2)	0.22907 (14)	0.48755 (6)	0.0406 (3)	
H1WA	1.219 (4)	0.1475 (17)	0.4739 (11)	0.061*	
H1WB	1.322 (4)	0.277 (2)	0.4726 (11)	0.061*	
O1	0.4310 (2)	0.35356 (13)	0.35586 (6)	0.0390 (3)	
O2	0.9399 (2)	0.53680 (13)	0.41861 (6)	0.0413 (3)	
O3	0.6857 (2)	0.36827 (14)	0.46300 (6)	0.0410 (3)	
O4	0.2323 (2)	0.12098 (13)	0.64264 (6)	0.0377 (3)	
O5	0.7730 (2)	0.03976 (14)	0.54726 (6)	0.0434 (3)	
O6	0.6637 (2)	0.23592 (12)	0.59560 (5)	0.0341 (2)	

### Atomic displacement parameters (Å^2^)

**Table:**

	*U*^11^	*U*^22^	*U*^33^	*U*^12^	*U*^13^	*U*^23^
Zn1	0.03023 (10)	0.03378 (11)	0.03012 (12)	−0.00814 (7)	0.00225 (7)	−0.00527 (7)
C1	0.0373 (9)	0.0404 (9)	0.0432 (11)	−0.0064 (7)	−0.0025 (7)	0.0008 (7)
C2	0.0376 (9)	0.0432 (10)	0.0552 (12)	−0.0100 (7)	−0.0072 (8)	−0.0061 (8)
C3	0.0369 (9)	0.0397 (9)	0.0450 (11)	0.0040 (7)	−0.0095 (8)	−0.0097 (7)
C4	0.0491 (11)	0.0586 (13)	0.0602 (14)	0.0003 (9)	−0.0201 (10)	−0.0150 (10)
C5	0.0667 (14)	0.0770 (16)	0.0483 (14)	0.0087 (12)	−0.0250 (11)	−0.0167 (11)
C6	0.0645 (13)	0.0748 (15)	0.0369 (11)	0.0114 (11)	−0.0087 (10)	−0.0039 (10)
C7	0.0519 (11)	0.0571 (12)	0.0402 (11)	0.0015 (9)	−0.0059 (9)	−0.0018 (9)
C8	0.0367 (8)	0.0381 (9)	0.0366 (10)	0.0059 (7)	−0.0056 (7)	−0.0055 (7)
C9	0.0356 (8)	0.0381 (9)	0.0398 (10)	−0.0048 (7)	−0.0052 (7)	−0.0010 (7)
C10	0.0306 (8)	0.0329 (8)	0.0367 (9)	0.0002 (6)	−0.0067 (7)	−0.0043 (6)
C11	0.0346 (8)	0.0290 (8)	0.0343 (9)	−0.0056 (6)	−0.0021 (7)	−0.0021 (6)
C12	0.0308 (7)	0.0290 (8)	0.0326 (9)	−0.0006 (6)	0.0015 (6)	−0.0076 (6)
C13	0.0328 (8)	0.0417 (9)	0.0407 (10)	−0.0001 (7)	0.0045 (7)	−0.0074 (7)
C14	0.0378 (9)	0.0382 (9)	0.0407 (10)	−0.0063 (7)	0.0025 (7)	−0.0019 (7)
C15	0.0498 (11)	0.0569 (12)	0.0500 (13)	0.0033 (9)	−0.0046 (9)	−0.0030 (9)
C16	0.0630 (13)	0.0693 (15)	0.0483 (13)	−0.0044 (11)	−0.0131 (10)	0.0052 (10)
C17	0.0758 (15)	0.0727 (15)	0.0358 (11)	−0.0136 (12)	0.0002 (10)	−0.0069 (10)
C18	0.0623 (13)	0.0606 (13)	0.0422 (12)	−0.0017 (10)	0.0060 (10)	−0.0106 (9)
C19	0.0460 (10)	0.0408 (10)	0.0382 (10)	−0.0057 (7)	0.0059 (8)	−0.0041 (7)
C20	0.0475 (10)	0.0447 (10)	0.0450 (11)	0.0079 (8)	0.0085 (8)	−0.0076 (8)
C21	0.0381 (9)	0.0416 (10)	0.0404 (10)	0.0032 (7)	0.0047 (7)	0.0000 (7)
C22	0.0314 (8)	0.0342 (8)	0.0342 (9)	−0.0075 (6)	0.0073 (7)	−0.0025 (6)
C23	0.0368 (8)	0.0325 (8)	0.0328 (9)	−0.0068 (6)	0.0054 (7)	−0.0046 (6)
C24	0.0303 (7)	0.0320 (8)	0.0255 (8)	−0.0037 (6)	−0.0005 (6)	−0.0016 (6)
O1W	0.0385 (6)	0.0351 (6)	0.0486 (8)	−0.0080 (5)	0.0147 (5)	−0.0075 (5)
O1	0.0411 (6)	0.0423 (7)	0.0334 (7)	−0.0143 (5)	−0.0084 (5)	0.0028 (5)
O2	0.0434 (7)	0.0410 (7)	0.0410 (7)	−0.0147 (5)	−0.0017 (5)	−0.0112 (5)
O3	0.0349 (6)	0.0536 (7)	0.0338 (7)	−0.0106 (5)	−0.0038 (5)	0.0046 (5)
O4	0.0314 (6)	0.0479 (7)	0.0342 (7)	−0.0015 (5)	0.0050 (5)	−0.0069 (5)
O5	0.0391 (6)	0.0463 (7)	0.0464 (8)	−0.0059 (5)	0.0126 (5)	−0.0160 (5)
O6	0.0368 (6)	0.0306 (6)	0.0350 (6)	−0.0078 (4)	0.0043 (5)	−0.0035 (5)

### Geometric parameters (Å, °)

**Table:**

Zn1—O2^i^	1.9492 (12)	C12—O2	1.2518 (19)
Zn1—O3	2.0143 (12)	C13—C22	1.366 (2)
Zn1—O6	1.9567 (11)	C13—C14	1.416 (3)
Zn1—O1W	1.9496 (12)	C13—H13A	0.9300
Zn1—C24	2.5875 (15)	C14—C15	1.414 (3)
C1—C2	1.359 (3)	C14—C19	1.418 (3)
C1—C10	1.410 (2)	C15—C16	1.362 (3)
C1—H1A	0.9300	C15—H15A	0.9300
C2—C3	1.409 (3)	C16—C17	1.402 (3)
C2—H2A	0.9300	C16—H16A	0.9300
C3—C8	1.412 (3)	C17—C18	1.362 (3)
C3—C4	1.420 (3)	C17—H17A	0.9300
C4—C5	1.354 (3)	C18—C19	1.415 (3)
C4—H4A	0.9300	C18—H18A	0.9300
C5—C6	1.395 (3)	C19—C20	1.415 (3)
C5—H5A	0.9300	C20—C21	1.357 (3)
C6—C7	1.362 (3)	C20—H20A	0.9300
C6—H6A	0.9300	C21—C22	1.415 (2)
C7—C8	1.408 (3)	C21—H21A	0.9300
C7—H7A	0.9300	C22—O4	1.373 (2)
C8—C9	1.418 (2)	C23—O4	1.4183 (19)
C9—C10	1.363 (2)	C23—C24	1.511 (2)
C9—H9A	0.9300	C23—H23A	0.9700
C10—O1	1.3712 (19)	C23—H23B	0.9700
C11—O1	1.4201 (18)	C24—O5	1.2430 (19)
C11—C12	1.496 (2)	C24—O6	1.2609 (19)
C11—H11A	0.9700	O1W—H1WA	0.821 (15)
C11—H11B	0.9700	O1W—H1WB	0.791 (15)
C12—O3	1.251 (2)	O2—Zn1^i^	1.9492 (11)
			
O2^i^—Zn1—O1W	105.14 (5)	C22—C13—H13A	120.0
O2^i^—Zn1—O6	99.93 (5)	C14—C13—H13A	120.0
O1W—Zn1—O6	138.94 (5)	C15—C14—C13	121.90 (17)
O2^i^—Zn1—O3	115.25 (5)	C15—C14—C19	118.50 (18)
O1W—Zn1—O3	102.70 (6)	C13—C14—C19	119.59 (16)
O6—Zn1—O3	95.10 (5)	C16—C15—C14	121.0 (2)
O2^i^—Zn1—C24	124.97 (5)	C16—C15—H15A	119.5
O1W—Zn1—C24	113.10 (5)	C14—C15—H15A	119.5
O6—Zn1—C24	28.08 (5)	C15—C16—C17	120.4 (2)
O3—Zn1—C24	93.56 (5)	C15—C16—H16A	119.8
C2—C1—C10	120.29 (17)	C17—C16—H16A	119.8
C2—C1—H1A	119.9	C18—C17—C16	120.4 (2)
C10—C1—H1A	119.9	C18—C17—H17A	119.8
C1—C2—C3	121.32 (17)	C16—C17—H17A	119.8
C1—C2—H2A	119.3	C17—C18—C19	120.7 (2)
C3—C2—H2A	119.3	C17—C18—H18A	119.6
C2—C3—C8	118.28 (16)	C19—C18—H18A	119.6
C2—C3—C4	122.66 (18)	C18—C19—C20	122.73 (18)
C8—C3—C4	119.04 (19)	C18—C19—C14	119.02 (18)
C5—C4—C3	120.6 (2)	C20—C19—C14	118.22 (17)
C5—C4—H4A	119.7	C21—C20—C19	121.59 (17)
C3—C4—H4A	119.7	C21—C20—H20A	119.2
C4—C5—C6	120.4 (2)	C19—C20—H20A	119.2
C4—C5—H5A	119.8	C20—C21—C22	119.80 (17)
C6—C5—H5A	119.8	C20—C21—H21A	120.1
C7—C6—C5	120.6 (2)	C22—C21—H21A	120.1
C7—C6—H6A	119.7	C13—C22—O4	126.08 (15)
C5—C6—H6A	119.7	C13—C22—C21	120.64 (16)
C6—C7—C8	120.9 (2)	O4—C22—C21	113.23 (15)
C6—C7—H7A	119.6	O4—C23—C24	113.14 (13)
C8—C7—H7A	119.6	O4—C23—H23A	109.0
C7—C8—C3	118.54 (17)	C24—C23—H23A	109.0
C7—C8—C9	121.79 (17)	O4—C23—H23B	109.0
C3—C8—C9	119.67 (17)	C24—C23—H23B	109.0
C10—C9—C8	120.19 (16)	H23A—C23—H23B	107.8
C10—C9—H9A	119.9	O5—C24—O6	122.57 (14)
C8—C9—H9A	119.9	O5—C24—C23	118.81 (14)
C9—C10—O1	125.40 (15)	O6—C24—C23	118.57 (14)
C9—C10—C1	120.21 (16)	O5—C24—Zn1	76.17 (9)
O1—C10—C1	114.38 (15)	O6—C24—Zn1	46.93 (7)
O1—C11—C12	110.15 (13)	C23—C24—Zn1	162.49 (11)
O1—C11—H11A	109.6	Zn1—O1W—H1WA	124.7 (15)
C12—C11—H11A	109.6	Zn1—O1W—H1WB	123.9 (16)
O1—C11—H11B	109.6	H1WA—O1W—H1WB	110.2 (19)
C12—C11—H11B	109.6	C10—O1—C11	116.83 (13)
H11A—C11—H11B	108.1	C12—O2—Zn1^i^	141.63 (11)
O3—C12—O2	124.83 (15)	C12—O3—Zn1	120.45 (10)
O3—C12—C11	120.08 (14)	C22—O4—C23	118.83 (13)
O2—C12—C11	115.07 (14)	C24—O6—Zn1	104.99 (10)
C22—C13—C14	120.06 (16)		

Symmetry codes: (i) −*x*+2, −*y*+1, −*z*+1.

### Hydrogen-bond geometry (Å, °)

**Table:**

*D*—H···*A*	*D*—H	H···*A*	*D*···*A*	*D*—H···*A*
O1W—H1WA···O5^ii^	0.82 (2)	1.81 (2)	2.6284 (18)	174 (2)
O1W—H1WB···O1^iii^	0.79 (2)	2.53 (2)	3.1087 (17)	131 (2)
O1W—H1WB···O3^iii^	0.79 (2)	2.12 (2)	2.8724 (16)	158 (2)

Symmetry codes: (ii) −*x*+2, −*y*, −*z*+1; (iii) *x*+1, *y*, *z*.
